# Temperature-Responsive *Antheraea pernyi* Silk Fibroin/Poly(N-isopropylacrylamide) Hydrogels for Controlled Protein Drug Delivery

**DOI:** 10.3390/gels12100888

**Published:** 2026-10-01

**Authors:** Boxiang Wang, Hangdan Xu, Hong Huang, Jinyang Xie, Huihui Liu

**Affiliations:** 1 Liaoning Provincial Key Laboratory of Functional Textile Materials, Liaodong University, Dandong 118001, China; 2 School of Textiles and Apparel, Liaodong University, Dandong 118001, China

**Keywords:** *Antheraea pernyi* silk fibroin, temperature-responsive hydrogel, PNIPAAm, lower critical solution temperature, drug delivery, controlled release, swelling behavior

## Abstract

Temperature-responsive hydrogels provide hydrated matrices for regulating protein release near physiological temperature. Building on a previously developed p(ASF-AGE-NIPAAm) hydrogel platform, this study evaluated five formulations with ASF-AGE feed contents of 10.0–57.1 wt% as protein delivery carriers. The hydrogels exhibited lower critical solution temperatures (LCSTs) of 31.8–33.0 °C and reversible swelling–deswelling transitions during cyclic temperature switching between 20 and 40 °C. Increasing the ASF-AGE content decreased the equilibrium swelling ratio from 17.3 to 10.6 g/g, while the bovine serum albumin (BSA) encapsulation efficiency varied between 73.5% and 76.8% and the drug loading capacity decreased from 452.6 to 380.2 mg/g. BSA release was evaluated in phosphate-buffered saline (PBS, pH 7.4) and depended on both temperature and formulation: the apparent cumulative release at the end of the observation period ranged from 37.6% to 54.1% at 20 °C and from 38.4% to 61.2% at 37 °C. Release was higher at 37 °C, coinciding with thermally induced network contraction, and ASF-rich formulations generally released less BSA. These Results define a composition–property–release relationship for p(ASF-AGE-NIPAAm) hydrogels and show that the ASF content can be adjusted to balance protein loading against temperature-responsive release.

## 1. Introduction

Stimuli-responsive hydrogels, often termed “smart” hydrogels, are three-dimensional polymeric networks that undergo reversible volume phase transitions or gel–sol transformations in response to environmental triggers such as temperature, pH, light, or biochemical signals [1]. Recent biomedical hydrogel research has increasingly focused on hydrated networks for controlled delivery and tissue-regeneration scaffolds [2]. Among the available stimuli, temperature is attractive for biomedical use because it is easy to control, non-invasive, and directly relevant to physiological conditions [3,4]. Temperature-responsive hydrogels have been investigated for controlled drug delivery, tissue engineering, cell encapsulation, and wound healing, where temperature changes can modulate release kinetics and scaffold properties [5,6].

Among synthetic temperature-responsive polymers, poly(N-isopropylacrylamide) (PNIPAAm) and its derivatives have received the most attention [7,8]. PNIPAAm exhibits a well-defined lower critical solution temperature (LCST) of approximately 32 °C in aqueous solution, close to human body temperature (37 °C), and this temperature can be tuned by copolymerization with hydrophilic or hydrophobic comonomers [9]. Below the LCST, PNIPAAm chains adopt an extended hydrophilic conformation stabilized by hydrogen bonding between amide groups and water; above the LCST, these interactions are disrupted and hydrophobic association of the isopropyl groups drives chain collapse and volume shrinkage [10,11]. This reversible phase transition underlies the use of PNIPAAm-based hydrogels as injectable or implantable delivery systems whose release can be triggered or modulated by physiological or externally applied temperature changes [12].

Protein therapeutics require carriers that maintain hydration, preserve protein stability, and allow release to be regulated without harsh processing conditions. Recent work on protein delivery has produced nanoscale platforms for efficient cytosolic transport of protein therapeutics [13]; temperature-responsive hydrogels offer a complementary route, serving as local hydrated reservoirs whose release can be modulated by thermally induced swelling or contraction. Bovine serum albumin (BSA) was selected here as a model protein because it is stable, well characterized, and commonly used to evaluate protein loading and release.

Despite their useful thermoresponsive behavior, PNIPAAm-based hydrogels face limitations that restrict clinical translation, including relatively poor mechanical properties, limited biodegradability, insufficient control over protein loading, and concerns over a non-degradable synthetic backbone [14,15]. Incorporating natural biopolymers into PNIPAAm-based networks is a common strategy for combining synthetic thermoresponsiveness with the biocompatibility and biodegradability of naturally derived materials [16,17].

Silk fibroin (SF), a natural fibrous protein produced by silkworms, has been investigated extensively as a biomaterial because of its biocompatibility, tunable biodegradability, mechanical performance, and processability [18,19]. Bombyx mori silk fibroin (BSF) has a long processing history and has been used in tissue engineering scaffolds, wound dressings, and drug delivery carriers [20,21]. Antheraea pernyi silk fibroin (ASF), by comparison, contains alanine-rich regions and the arginine-glycine-aspartate (RGD) tripeptide sequence, which is absent from BSF and provides a recognition motif for integrin-mediated cell adhesion [22,23,24,25,25]. BSF offers a mature materials platform with robust mechanical performance, whereas ASF adds intrinsic cell-adhesive functionality that may benefit cell-interactive scaffolds and protein delivery. For both proteins, mechanical stability and degradation depend strongly on processing, β-sheet content, and the surrounding biological environment [26,27].

ASF is rich in alanine (approximately 43%), glycine (approximately 27%), and serine (approximately 11%), and its secondary structure can contain β-sheet crystalline domains [28]. These features give mechanical stability and offer sites for polar and hydrophobic interactions with loaded proteins. The reactive amino and hydroxyl groups along ASF chains also permit chemical modification and covalent incorporation into hybrid hydrogel networks [29].

Several silk fibroin/PNIPAAm hybrid systems have been reported. Wang et al. developed regenerated ASF/PNIPAAm thermosensitive composite hydrogels with improved mechanical strength [29]. In our previous study, p(ASF-AGE-NIPAAm) hydrogels were synthesized through silk fibroin self-assembly-induced physical crosslinking without a conventional chemical crosslinker, and their thermoresponsive properties, enzymatic degradability, and cytocompatibility were established [30]. Gil et al. prepared PNIPAAm/silk interpenetrating or macroporous hybrid hydrogels and examined their swelling/deswelling, rheological, and actuation behavior [31,32]. These studies established the feasibility of combining silk fibroin with PNIPAAm, but protein loading and temperature-dependent release have not been compared across ASF-AGE compositions in a systematic manner.

The present study extends the previously developed p(ASF-AGE-NIPAAm) platform toward protein drug delivery. Its novelty lies not in the initial synthesis of the hydrogel, but in establishing how the ASF-AGE feed content relates to thermoresponsive swelling/deswelling, BSA loading, apparent release, and empirical release kinetics. To this end, the study examines: (i) dynamic and equilibrium swelling at different temperatures; (ii) deswelling kinetics and reversible temperature responsiveness; (iii) BSA encapsulation efficiency and loading capacity; and (iv) temperature-dependent apparent release and model-dependent release kinetics. The resulting composition–property–release analysis is intended to support the further optimization of ASF/PNIPAAm-based temperature-responsive protein carriers.

## 2. Results and Discussion

### 2.1. Swelling Morphology

**Figure 1:**
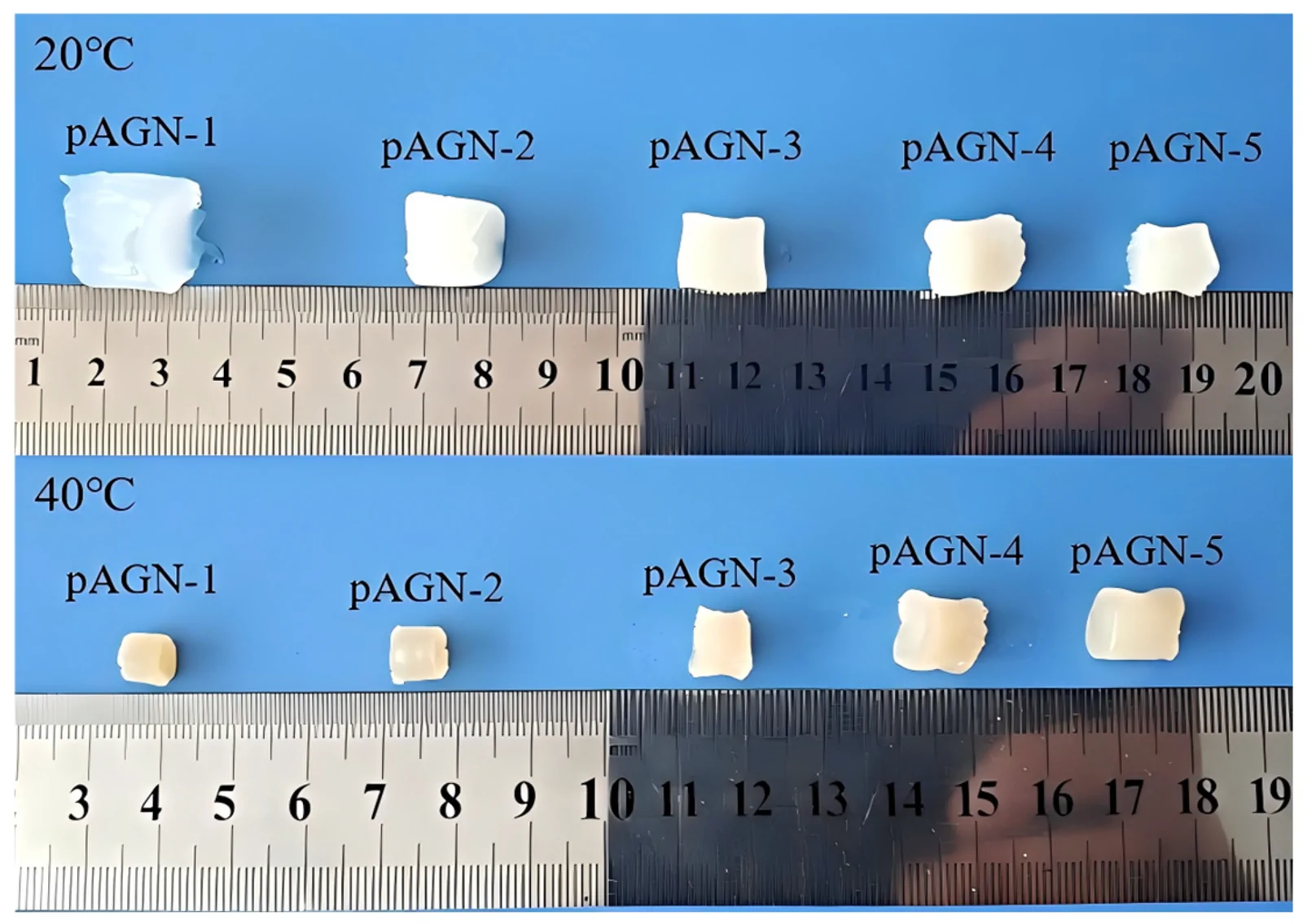
The swelling morphology of the hydrogel samples after reaching swelling equilibrium at 20 °C and 40 °C.

The macroscopic morphological changes in the p(ASF-AGE-NIPAAm) hydrogels upon swelling at different temperatures were recorded using digital photography to provide a visual assessment of their thermally induced volume transitions. Figure 1 presents the photographs of the hydrogel samples at 20 °C (below the LCST) and 40 °C (above the LCST) after reaching swelling equilibrium.

At 20 °C, all hydrogel samples exhibited substantial volume expansion compared to their dry state, indicating a hydrated, swollen character in the low-temperature regime. As the ASF-AGE feed content increased from 10.0 wt% (pAGN-1) to 57.1 wt% (pAGN-5), the extent of volumetric expansion progressively decreased. This observation is consistent with restricted swelling capacity at higher ASF-AGE incorporation.

When the swelling medium was heated to 40 °C, all samples contracted as a result of the thermally induced phase transition of the PNIPAAm component. The overall appearance and shape of the hydrogels remained essentially unchanged, indicating that the network stayed intact during deswelling. The contraction was more pronounced in hydrogels with lower ASF-AGE content: pAGN-1 shrank the most, whereas pAGN-5 showed relatively modest shrinkage.

These morphological trends are consistent with two effects of increasing ASF content. Higher ASF loading may promote intermolecular associations within the composite network and increase resistance to network expansion. The alanine-rich, relatively hydrophobic sequences of ASF may also reduce the overall hydrophilicity of the composite hydrogel and constrain the relaxation of PNIPAAm chains. These two effects together may account for the lower swelling observed at higher ASF contents.

### 2.2. Swelling Behavior

**Figure 2:**
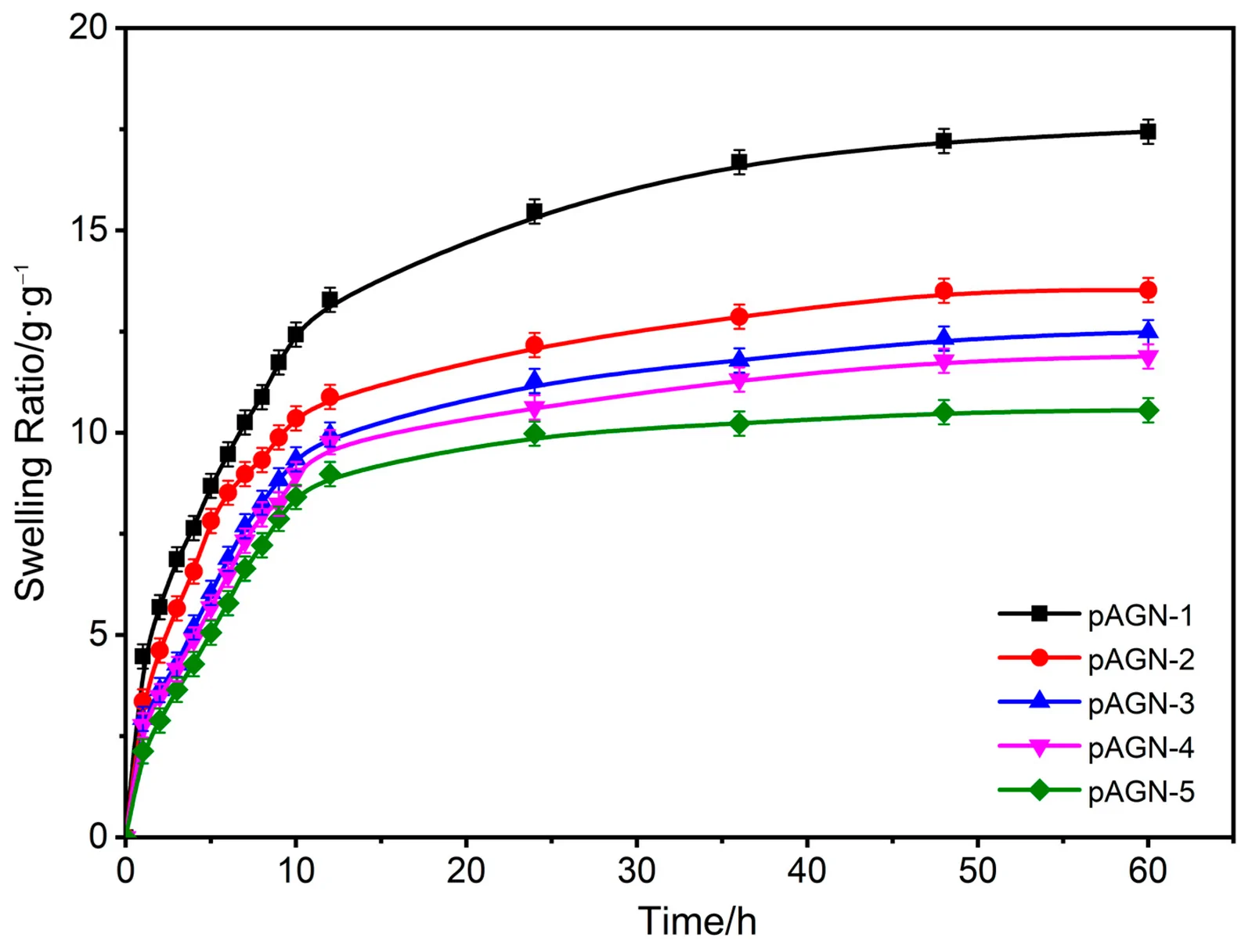
The dynamic swelling profiles of the p(ASF-AGE-NIPAAm) hydrogels in 0.03 M PBS at 20 °C.

The dynamic swelling profiles of the p(ASF-AGE-NIPAAm) hydrogels in 0.03 M PBS at 20 °C are presented in Figure 2. All hydrogel formulations exhibited rapid initial water uptake, with the swelling ratio increasing sharply during the first several hours of immersion. The hydrogels reached approximately 80% of their equilibrium swelling ratio within 10 h, after which the swelling rate gradually decelerated. Beyond 30 h, the hydrogels approached swelling equilibrium, as evidenced by the plateau regions in the swelling curves.

The dynamic swelling process of the p(ASF-AGE-NIPAAm) hydrogels may be influenced by several coupled processes: diffusion of water into the three-dimensional polymer network, hydration of hydrophilic groups, relaxation and extension of polymer chains, and the osmotic pressure difference between the hydrogel interior and the external medium. Upon immersion of the dry gel in aqueous medium, water molecules diffuse into the network and interact with hydrophilic hydroxyl, carboxyl, and amide groups, leading to rapid water uptake. As swelling progresses, the osmotic pressure gradient gradually decreases, while the elastic retractive forces of the polymer network counterbalance the swelling pressure until equilibrium is approached.

The equilibrium swelling ratio depended on the ASF-AGE content and decreased progressively as the ASF-AGE/PNIPAAm ratio increased: pAGN-1 (10.0 wt% ASF-AGE) reached 17.3 g/g, whereas pAGN-5 (57.1 wt% ASF-AGE) reached only 10.6 g/g. This reduction may be associated with increased intermolecular associations within the ASF-rich composite network, the lower overall hydrophilicity arising from the alanine-rich sequences of ASF, and possible competition between ASF–PNIPAAm interactions and water–polymer hydrogen bonding.

### 2.3. Swelling Kinetics

The dynamic swelling process of a hydrogel is a complex phenomenon that can be conceptually divided into three sequential stages: (i) diffusion of water molecules into the three-dimensional polymer network; (ii) hydration of hydrophilic groups through hydrogen bonding between water molecules and polar functional groups on the polymer chains, leading to destabilization of interchain interactions; and (iii) relaxation and extension of the macromolecular chains in the aqueous medium, resulting in network expansion. The relative rates of water diffusion and polymer chain relaxation determine the overall swelling kinetics.

To analyze the swelling mechanism of the p(ASF-AGE-NIPAAm) hydrogels, the dynamic swelling data were interpreted using the Fickian diffusion/relaxation kinetic model, which has been widely employed to describe the swelling behavior of hydrogel systems. For the initial stage of swelling (up to approximately 60% of the equilibrium swelling ratio), the fractional swelling can be expressed by the following power-law relationship:

(1) $$\frac{M_{t}}{M_{e}}=kt^{n}$$

where M_t_ and M_e_ represent the swelling ratios at time t and at equilibrium, respectively, k is a characteristic constant related to the gel structure and swelling rate, and *n* is the swelling exponent indicative of the transport mechanism. Taking the logarithm of both sides yields a linear form suitable for data fitting:

(2) $$ln\frac{M_{t}}{M_{e}}=lnk+nlnt$$

According to the Fickian diffusion theory, the value of the exponent *n* discriminates among different transport regimes: when *n* ≤ 0.5, water diffusion is faster than polymer chain relaxation, and the swelling follows Fickian diffusion (diffusion-controlled); when 0.5 < *n* < 1, the swelling process is governed by both water diffusion and polymer chain relaxation in a comparable manner, corresponding to non-Fickian or anomalous transport; when *n* ≥ 1, polymer chain relaxation dominates over water diffusion, corresponding to Case-II transport.

**Figure 3:**
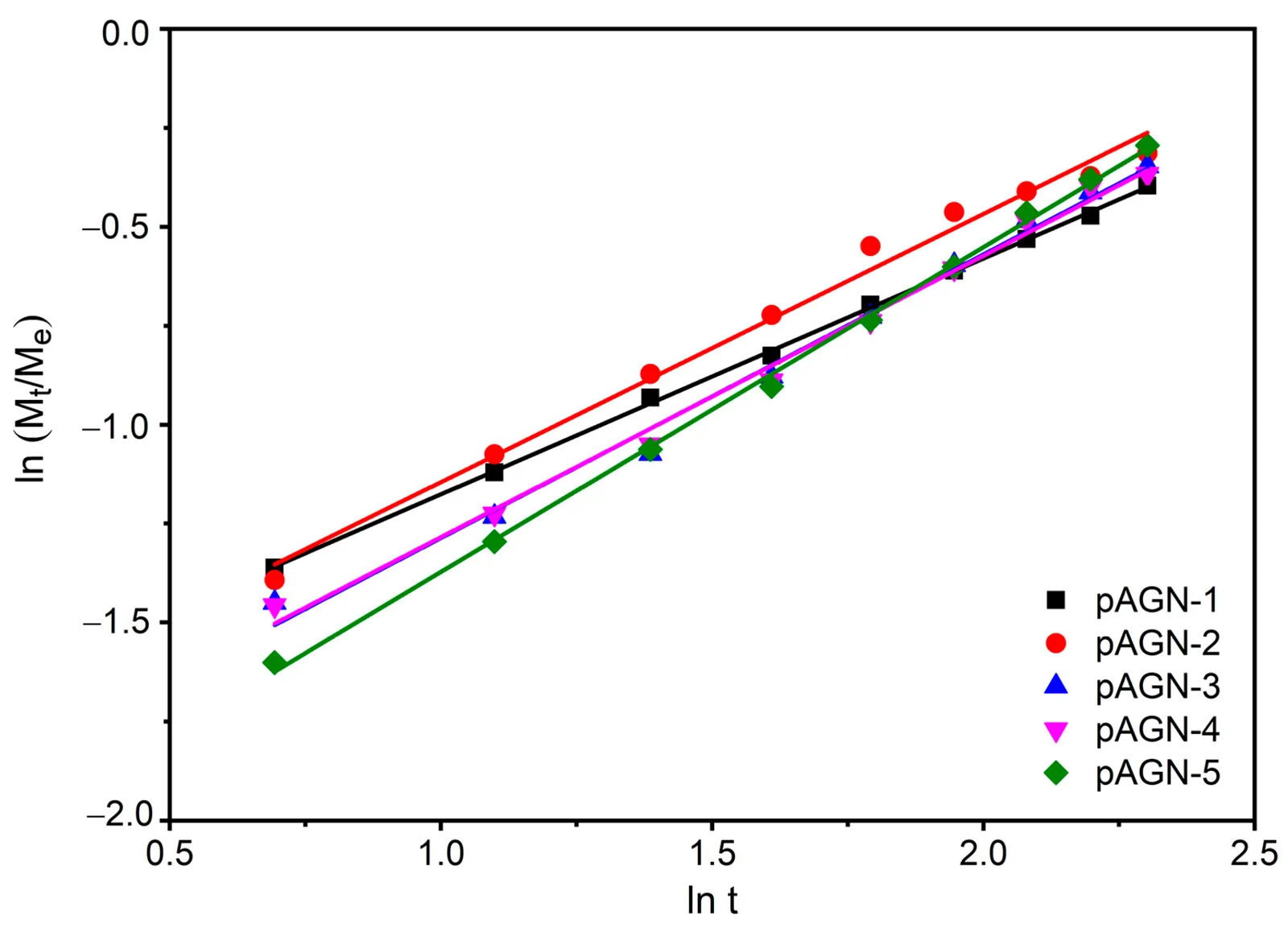
Linear fits of ln(M_t_/M_e_) versus ln t for p(ASF-AGE-NIPAAm) hydrogels.

The experimental swelling data were processed by plotting ln(M_t_/M_e_) against lnt, and the resulting linear fits are shown in Figure 3. The corresponding kinetic parameters (*n* values and correlation coefficients) are summarized in Table 1.

All fitted curves showed high linearity, with correlation coefficients (R^2^) exceeding 0.99, supporting the power-law model as an empirical description of the swelling kinetics. The *n* values of all five formulations fell between 0.5 and 1, indicating non-Fickian or anomalous transport. Swelling of the p(ASF-AGE-NIPAAm) hydrogels appears to involve coupled contributions from water diffusion and macromolecular chain relaxation rather than either process alone.

The *n* value increased gradually with ASF-AGE content, from 0.596 for pAGN-1 to 0.821 for pAGN-5, suggesting that polymer-chain relaxation contributes more to the swelling process at higher ASF contents. This trend is consistent with restricted segmental mobility in ASF-rich hydrogels, where rigid ASF-rich domains and possible ASF–PNIPAAm interactions may limit chain motion. As the ASF content increases, relaxation of the chain segments becomes increasingly rate-limiting, and the transport mechanism shifts toward the relaxation-dominated regime.

### 2.4. Deswelling Kinetics

**Figure 4:**
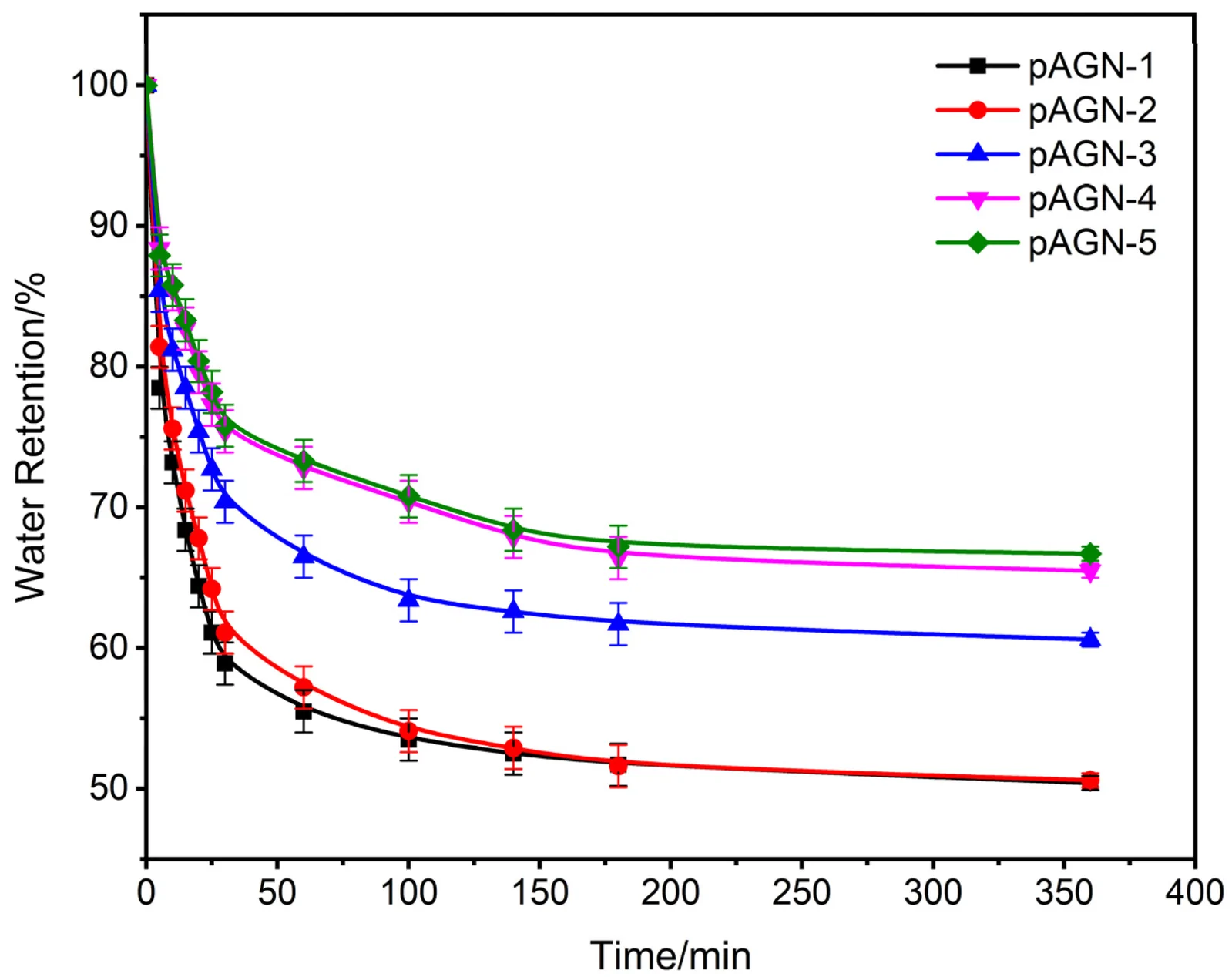
Water retention curves of p(ASF-AGE-NIPAAm) hydrogels as a function of time at 40 °C.

When an equilibrated swollen hydrogel is transferred from a low-temperature medium to a temperature above its LCST, the gel undergoes a phase transition-driven contraction accompanied by expulsion of water, a process referred to as deswelling. The rate of water loss during deswelling largely determines the response speed of a temperature-responsive hydrogel and its range of potential applications. To evaluate the deswelling behavior, hydrogel samples that had reached swelling equilibrium at 20 °C were rapidly transferred to a water bath at 40 °C, and the water retention was monitored as a function of time. The results are presented in Figure 4.

All hydrogel samples deswelled rapidly at 40 °C, as reflected by the sharp decline of the water retention (WR) curves (Figure 4). Deswelling was fastest during the initial 30 min; the WR then decreased more gradually and eventually levelled off. The equilibrium water retention depended strongly on composition: the residual WR values for pAGN-1 through pAGN-5 were 50.4%, 50.6%, 60.6%, 65.5%, and 66.7%, respectively. Hydrogels with higher PNIPAAm content expelled more water and reached lower retention, whereas ASF-rich hydrogels kept a larger fraction of the imbibed water—up to 66.7% for pAGN-5—and deswelled less.

These trends may be related to thermally induced deswelling in PNIPAAm-based hydrogels. When the temperature exceeds the LCST, hydrophobic association of the isopropyl groups promotes a coil-to-globule transition of PNIPAAm chains, accompanied by water expulsion. The higher equilibrium water retention of ASF-rich hydrogels may be associated with their lower PNIPAAm fraction, reduced hydration responsiveness, and restricted network contraction.

To further elucidate the deswelling mechanism, the deswelling kinetics were analyzed using a first-order rate equation. The linearized form of the equation is expressed as:

(3) $$ln\frac{M_{e}-M_{t}}{M_{e}-M_{d}}=-kt$$

where M_e_, M_d_, and M_t_ denote the equilibrium swollen mass, dry mass, and mass at time t, respectively, and k is the deswelling rate constant. A larger absolute value of k indicates a faster deswelling rate.

**Figure 5:**
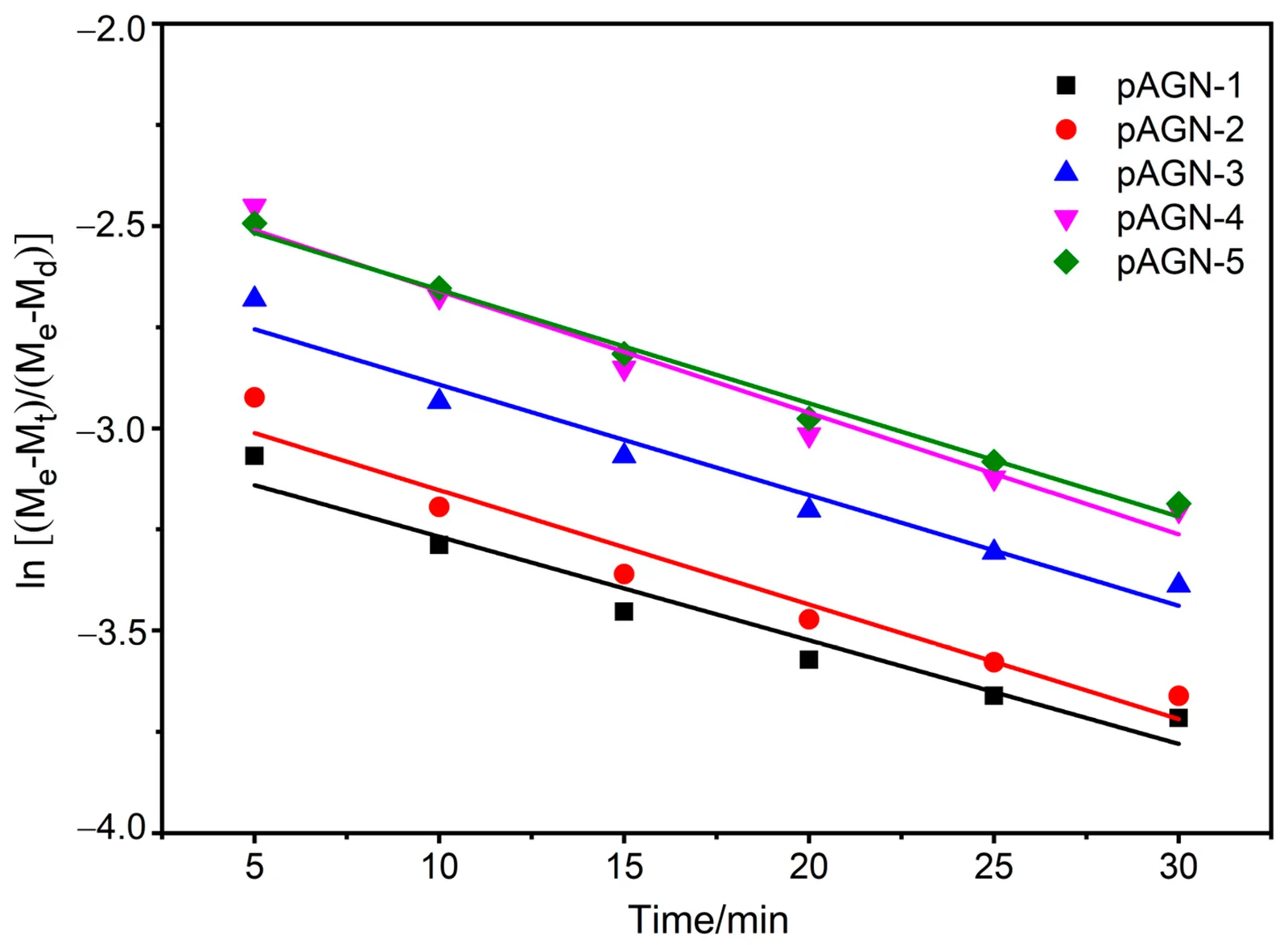
Linear fitting curves of ln[(M_e_ − M_t_)/(M_e_ − M_d_)] versus time for p(ASF-AGE-NIPAAm) hydrogels.

The plots of Equation (3) versus time are shown in Figure 5, and the corresponding kinetic parameters are compiled in Table 2.

All formulations exhibited satisfactory linear correlations (R^2^ > 0.93), indicating that the first-order model provides an empirical description of the deswelling process. The rate constants did not show a clear monotonic dependence on ASF content. However, the equilibrium water retention increased from 50.4% for pAGN-1 to 66.7% for pAGN-5, indicating that ASF-rich hydrogels retained more water after thermally induced contraction. This behavior may be associated with restricted network contraction and the presence of hydrophobic ASF-rich domains, which could slow water transport during deswelling.

### 2.5. Temperature-Responsive Behavior

PNIPAAm-based hydrogels are high-temperature-shrinkable temperature-responsive systems. Above a critical threshold—the LCST—the hydrogel remains in a contracted state; below this temperature, it exists in an expanded, swollen state. Below the LCST, PNIPAAm-based hydrogels are typically transparent and swollen. On heating above the LCST, the gel undergoes a sharp volume contraction accompanied by a fundamental change in the hydrophilic/hydrophobic character of the network, driven by the coil-to-globule transition of PNIPAAm chains. Measuring the equilibrium swelling ratio as a function of temperature gives direct access to the temperature responsiveness of the hydrogel and to its LCST.

**Figure 6:**
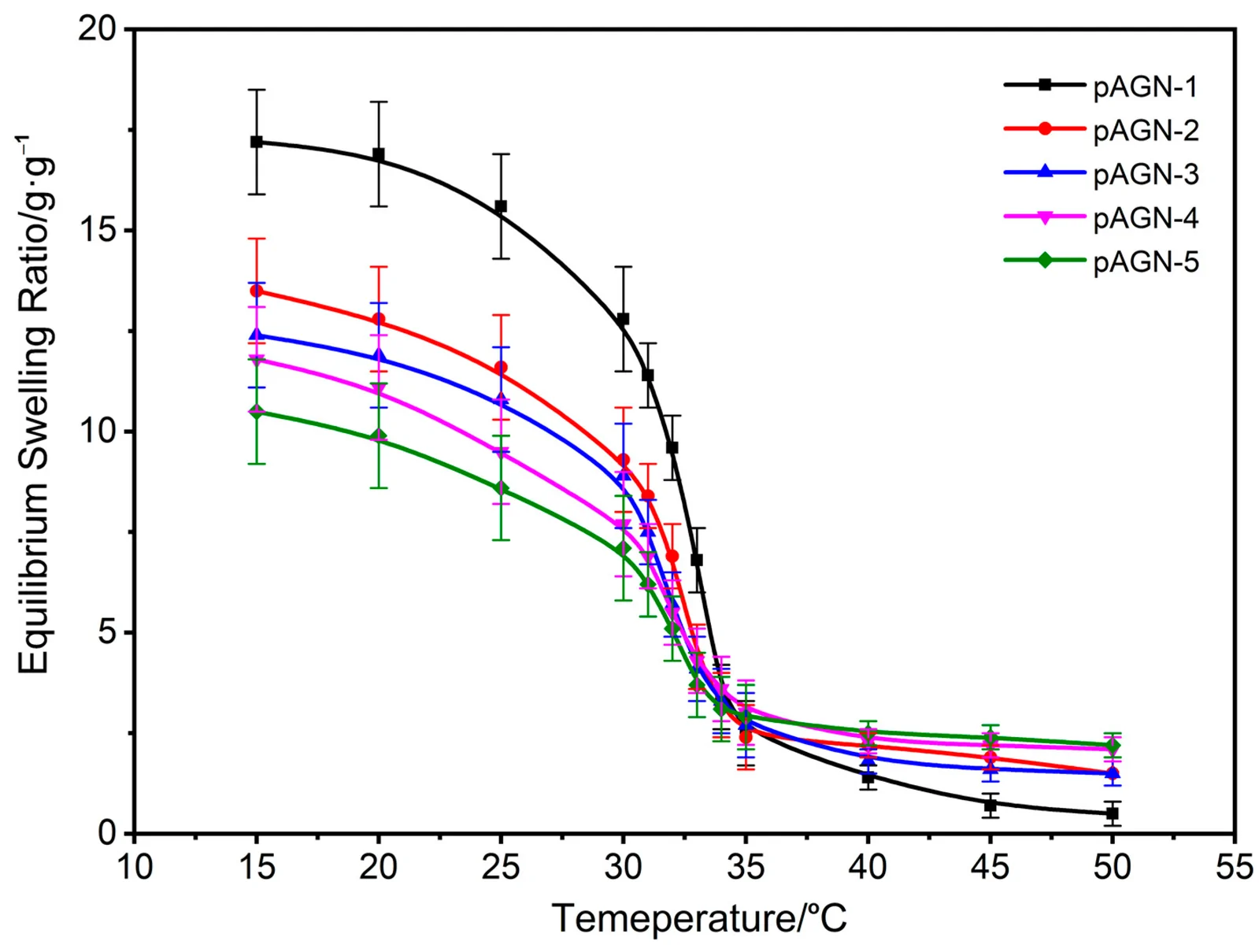
Equilibrium swelling ratio (ESR) curves of p(ASF-AGE-NIPAAm) hydrogels at various temperatures.

The equilibrium swelling ratios (ESR) of the p(ASF-AGE-NIPAAm) hydrogels measured across the temperature range of 15–50 °C are presented in Figure 6. All hydrogel formulations exhibited a qualitatively similar trend: the ESR gradually decreased as the temperature increased, reflecting the progressive dehydration and contraction of the PNIPAAm component. Below the LCST, water molecules are stabilized within the gel network through hydrogen bonding with the hydrophilic functional groups (amide groups) of PNIPAAm, resulting in a high equilibrium swelling ratio. As the temperature approaches and exceeds the LCST, thermal motion disrupts these hydrogen bonds, and the hydrophobic isopropyl groups become dominant, causing the polymer chains to adopt a collapsed conformation. Consequently, the gel network shrinks and a substantial fraction of the imbibed water is expelled through the gel pores, leading to a marked reduction in the equilibrium swelling ratio.

The differences in the absolute ESR values among the formulations may be associated with the varying ASF content. As the ASF content increases, more hydrophobic polyalanine sequences are incorporated into the network, which may reduce overall hydrogel hydrophilicity and lead to lower equilibrium swelling ratios across the temperature range. This interpretation is consistent with the observations discussed in Section 2.2.

**Figure 7:**
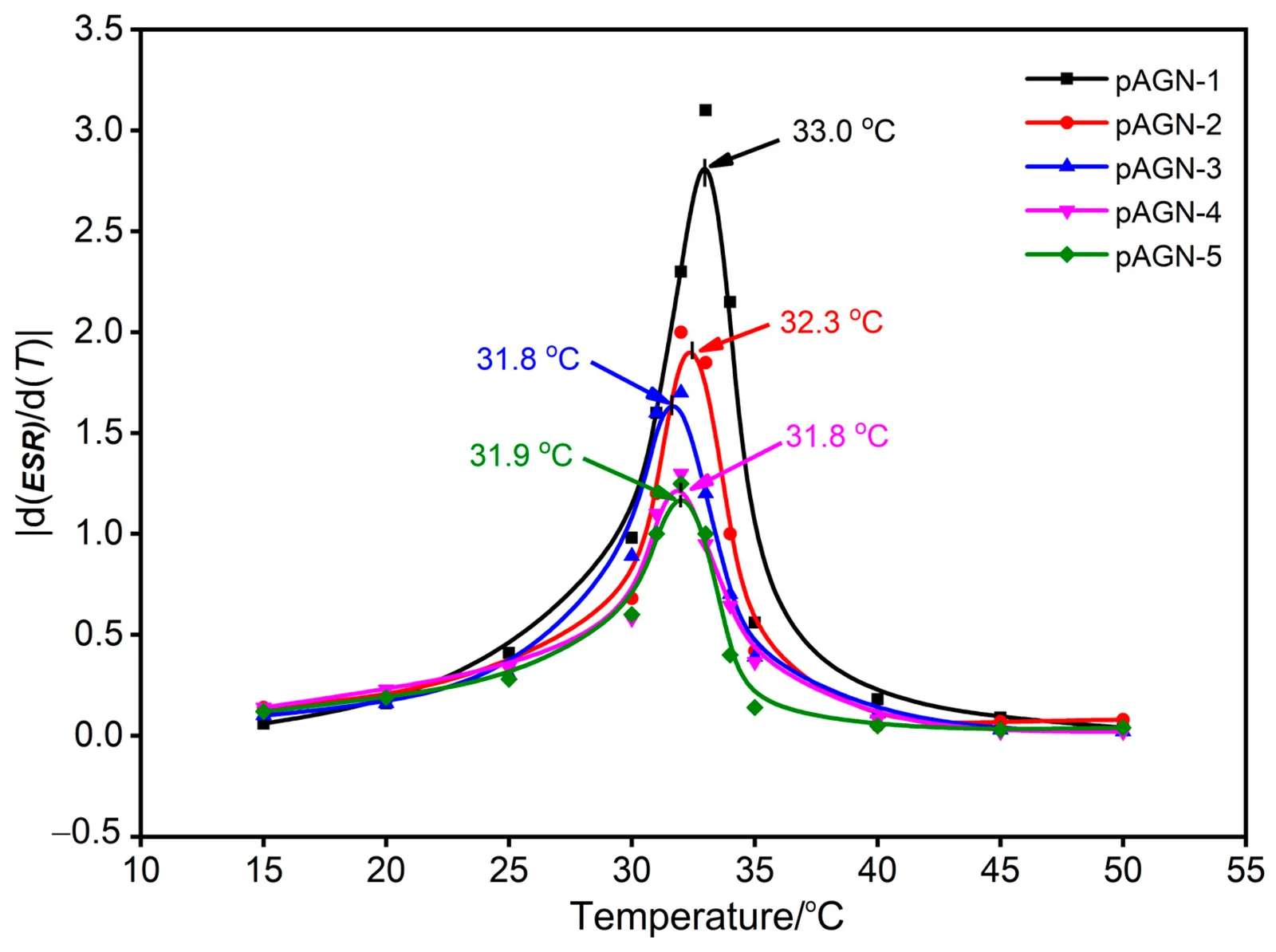
First-order derivative curves of the equilibrium swelling ratio (ESR) with respect to temperature for p(ASF-AGE-NIPAAm) hydrogels.

To precisely determine the LCST, the first-order derivative of the ESR with respect to temperature, (d(ESR)/d(T)), was calculated from the swelling data. The peak temperature corresponding to the maximum absolute value of the derivative identifies the LCST, while the peak area provides a measure of the responsiveness sensitivity of the hydrogel. The first-order derivative curves for all formulations are shown in Figure 7.

The derivative analysis revealed that the LCSTs of pAGN-1 through pAGN-5 were 33.0, 32.3, 31.8, 31.8, and 31.9 °C, respectively, all located in the vicinity of 32 °C and close to physiological temperature, which is favorable for biomedical applications. As the ASF content increased, the LCST exhibited a slight downward shift from 33.0 °C for pAGN-1 to 31.9 °C for pAGN-5, and the peak area of the derivative curves progressively decreased, indicating a reduction in the responsiveness sensitivity. This behavior can be explained by the combined effects of decreased hydrophilic group density and enhanced network rigidity: the incorporation of increasing amounts of ASF reduces the concentration of hydrophilic functional groups (amide groups from NIPAAm) in the network while simultaneously strengthening the physical entanglement between ASF and PNIPAAm chains. The slight decrease in LCST and the reduction in derivative peak area with increasing ASF-AGE content may reflect the decreased PNIPAAm fraction and the reduced hydration responsiveness of the ASF-rich formulations.

These results show that the temperature-responsive behavior of p(ASF-AGE-NIPAAm) hydrogels depends on both temperature and composition. Adjusting the ASF-AGE/PNIPAAm ratio tunes the LCST and the responsiveness, offering a route to temperature-triggered drug delivery systems with tailored phase-transition characteristics.

### 2.6. Pulsatile Temperature Response

**Figure 8:**
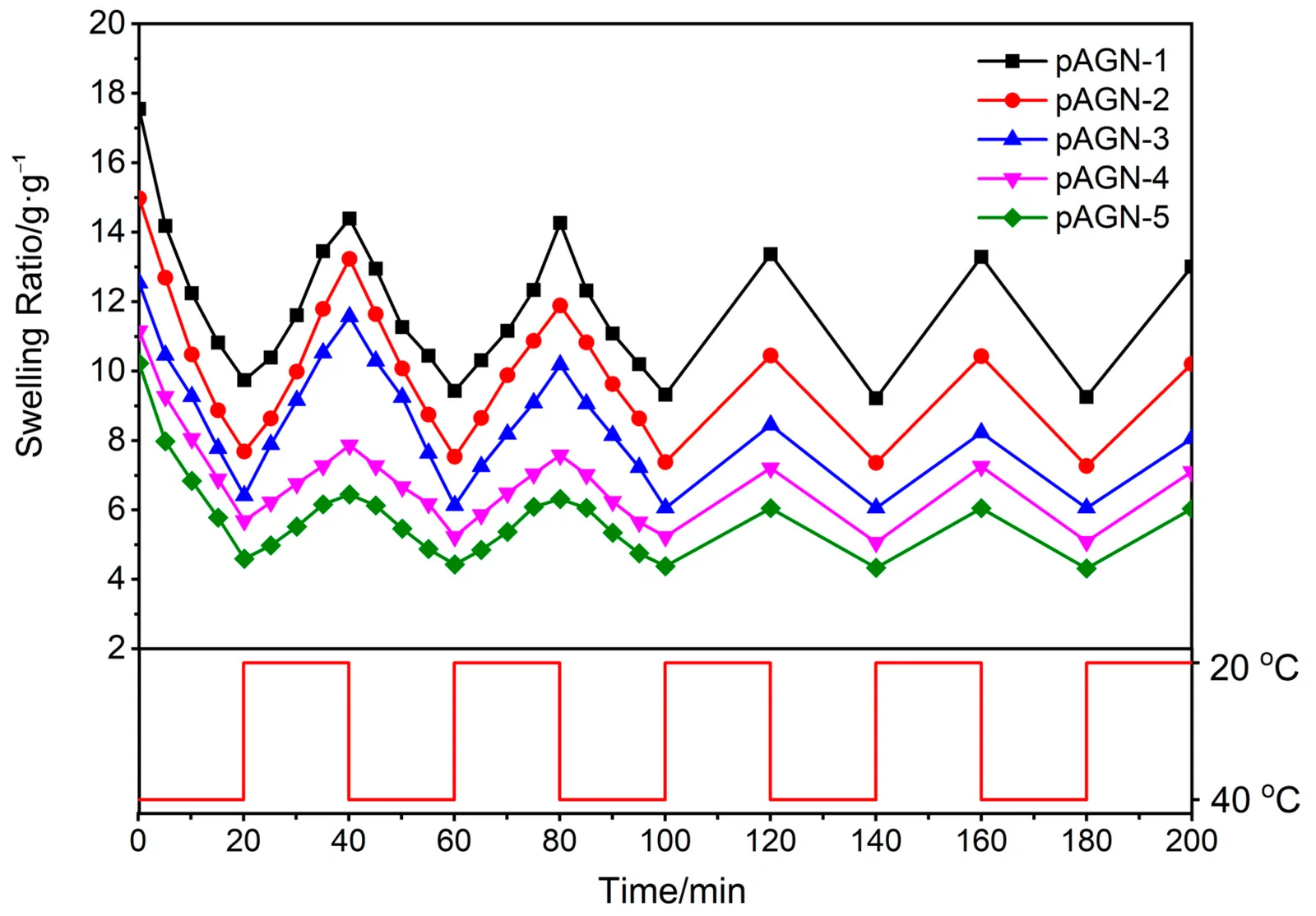
Dynamic swelling–deswelling curves of p(ASF-AGE-NIPAAm) hydrogels upon cyclic temperature switching between 20 °C and 40 °C.

For practical applications of stimuli-responsive hydrogels in drug delivery and actuation, the hydrogel must undergo reversible and reproducible swelling–deswelling transitions under repeated temperature switching. To evaluate the pulsatile temperature responsiveness of the p(ASF-AGE-NIPAAm) hydrogels, cyclic swelling–deswelling experiments were conducted by alternately immersing the hydrogel samples in PBS at 20 °C (below the LCST) and 40 °C (above the LCST), and the swelling ratio was measured at regular intervals. The results are presented in Figure 8.

All hydrogel formulations exhibited rapid and reversible volume transitions in response to the alternating temperature steps. When the temperature was switched from 20 °C to 40 °C, the gels underwent rapid contraction due to the PNIPAAm phase transition, with the initial deswelling occurring particularly quickly within the first few minutes. Upon returning the samples to 20 °C, the gels re-swelled as the PNIPAAm chains rehydrated and re-expanded. This cyclic pattern repeated consistently over multiple temperature cycles, demonstrating that all five hydrogel formulations possess good temperature-reversible responsive behavior.

Several noteworthy features emerged from the cyclic experiments. First, within each individual cycle, the hydrogels did not fully recover their original equilibrium swelling ratio during the reswelling phase following the deswelling step. As the number of cycles increased, the extent of swelling achievable during the low-temperature phase progressively decreased, indicating that the reswelling speed was considerably slower than the deswelling speed. This asymmetry between swelling and deswelling kinetics is characteristic of PNIPAAm-based hydrogels and has been attributed to the rapid formation of a dense, hydrophobic surface layer during the collapse phase, which hinders water re-entry during the reswelling phase.

The rate of swelling ratio decline across successive cycles was more pronounced for hydrogels with higher ASF content. This can be explained by the structural characteristics of ASF within the composite network. The faster decline in swelling ratio over successive cycles for ASF-rich hydrogels may be associated with slower rehydration and the presence of relatively hydrophobic ASF-rich domains, which could hinder water re-entry during the reswelling process. ASF is known to have a β-sheet-forming tendency, and this feature may contribute to the observed behavior.

Taken together, the p(ASF-AGE-NIPAAm) hydrogels exhibit favorable reversible temperature-responsive characteristics, with the ability to undergo repeated swelling–deswelling cycles upon temperature switching. The composition-dependent differences in the pulsatile response behavior provide additional design flexibility for controlling the dynamic performance of these hydrogels as temperature-triggered drug carriers.

### 2.7. BSA Quantification Curve

**Figure 9:**
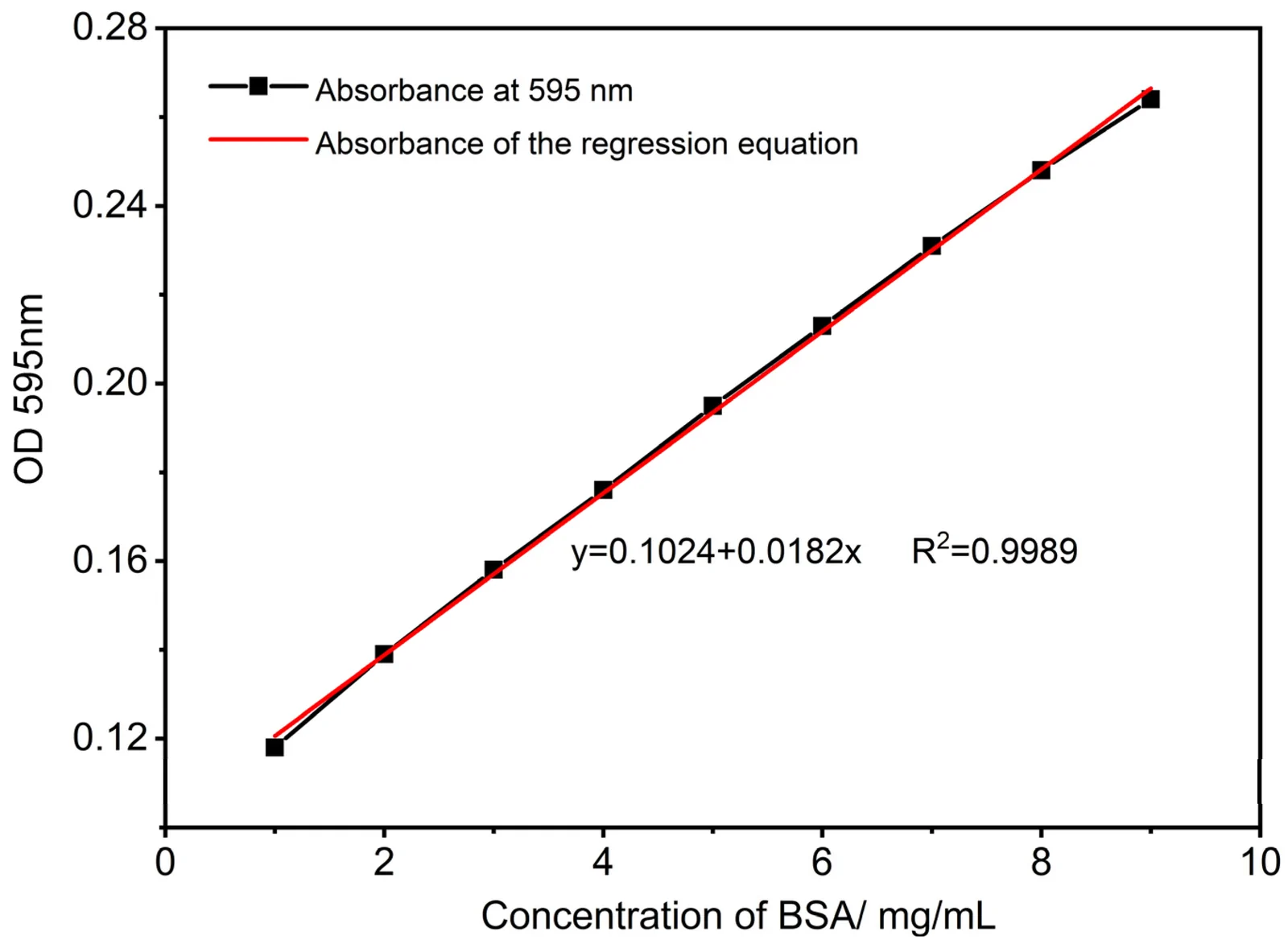
Calibration curve for BSA quantification using the Coomassie Brilliant Blue R250 assay.

BSA concentrations were estimated using the Coomassie Brilliant Blue R250 assay. OD595 denotes the absorbance measured at 595 nm. BSA calibration standards with concentrations ranging from 1 to 9 mg/mL were prepared in 0.03 M PBS and analyzed under the same assay conditions as the samples. The calibration curve (Figure 9) was expressed as y = 0.1024 + 0.0182x, where y represents OD595 and x represents the BSA concentration in mg/mL, with R^2^ = 0.9989. Unknown BSA concentrations were calculated using x = (y − 0.1024)/0.0182. The residual BSA concentrations in the loading supernatants, calculated from the encapsulation efficiencies, ranged from 1.16 to 1.33 mg/mL and were therefore within the established calibration range. In contrast, based on the loaded BSA amounts and the release-medium volume, the BSA concentrations in the release samples were below the lower limit of the calibration curve. Therefore, the release profiles are presented as apparent cumulative release profiles for comparing temperature- and composition-dependent release trends under identical assay conditions.

### 2.8. BSA Loading

The BSA loading experiments were conducted by immersing dry hydrogel samples in BSA solution and incubating at 25 °C with constant shaking for 5 days to reach equilibrium. The encapsulation efficiency (EE) and drug loading capacity (DL) of the five hydrogel formulations are summarized in Table 3.

As shown in Table 3, all five hydrogel samples achieved high encapsulation efficiencies, from 73.5 ± 1.3% for pAGN-1 to 76.8 ± 1.3% for pAGN-5, whereas the drug loading capacity ranged from 380 ± 17 mg/g for pAGN-5 to 453 ± 14 mg/g for pAGN-1. Two opposing trends emerged as the ASF content increased.

The encapsulation efficiency exhibited a slight but consistent increase as the ASF content increased from pAGN-1 to pAGN-5. This phenomenon can be attributed to specific molecular interactions between the ASF peptide chains and BSA molecules. The slight increase in encapsulation efficiency with increasing ASF content may be associated with favorable polar interactions between ASF peptide chains and BSA molecules, as suggested by previous studies on silk–protein interactions [31,32]. These hydrogen bonding interactions enhance the affinity between the hydrogel matrix and the protein drug, thereby promoting BSA retention within the network and contributing to the marginally improved encapsulation efficiency.

The drug loading capacity decreased progressively with increasing ASF content. The decrease in drug loading capacity with increasing ASF content is consistent with the lower swelling capacity and potentially greater transport resistance of ASF-rich hydrogels. Steric constraints within the composite network and possible BSA–ASF interactions may jointly influence the partitioning and diffusion of BSA during loading, which physically hinders the permeation and diffusion of BSA molecules into the interior regions of the hydrogel during the loading process [33]. Consequently, despite the favorable hydrogen bonding interactions, the steric constraints imposed by the tightened network ultimately result in reduced overall drug loading capacity. This trade-off between encapsulation efficiency and loading capacity highlights the importance of optimizing the ASF/NIPAAm ratio to balance protein affinity with network permeability for specific drug delivery applications.

### 2.9. BSA Release Behavior

**Figure 10:**
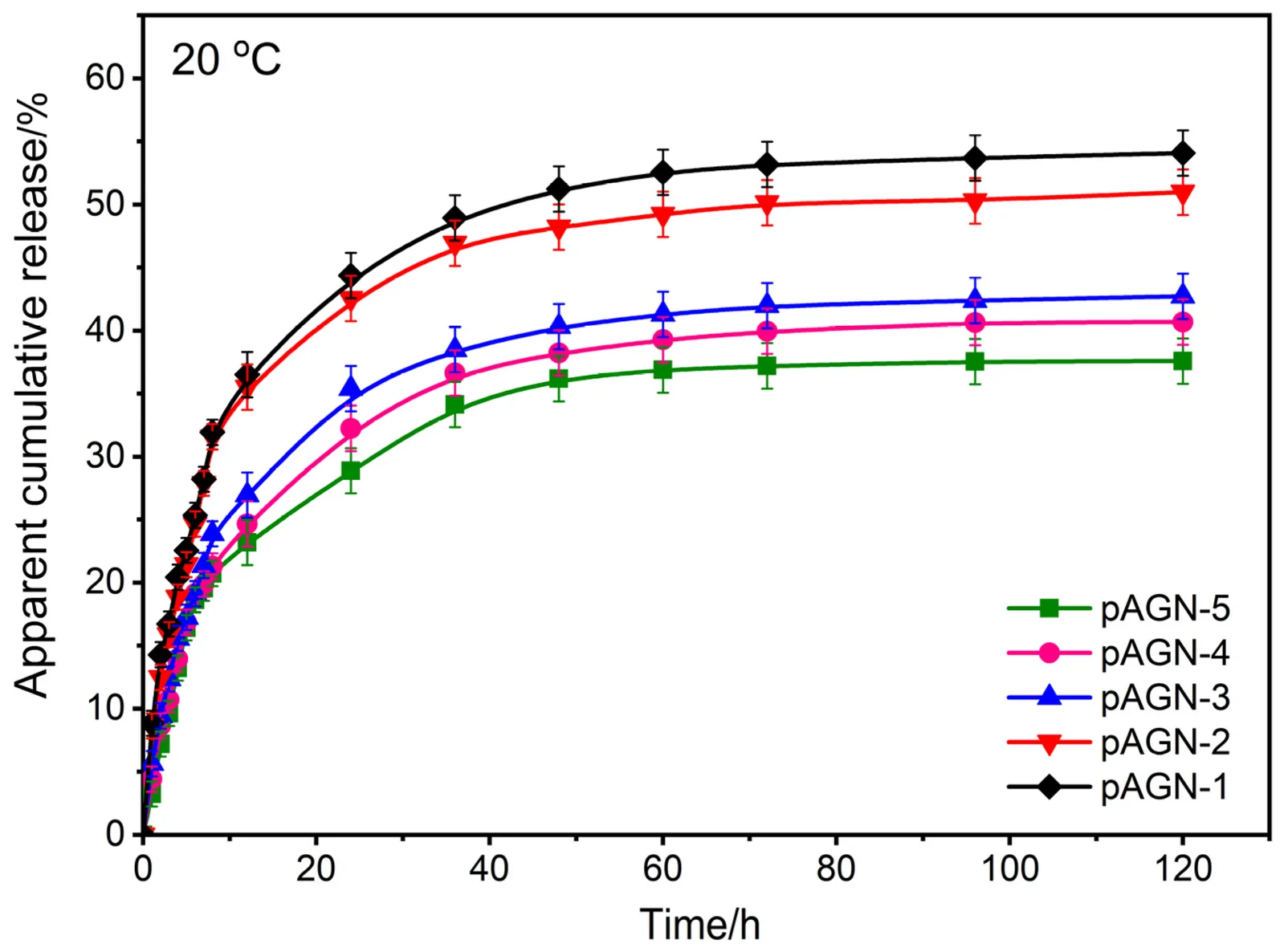
Apparent cumulative release curves of BSA from p(ASF-AGE-NIPAAm) hydrogels at 20 °C.

**Figure 11:**
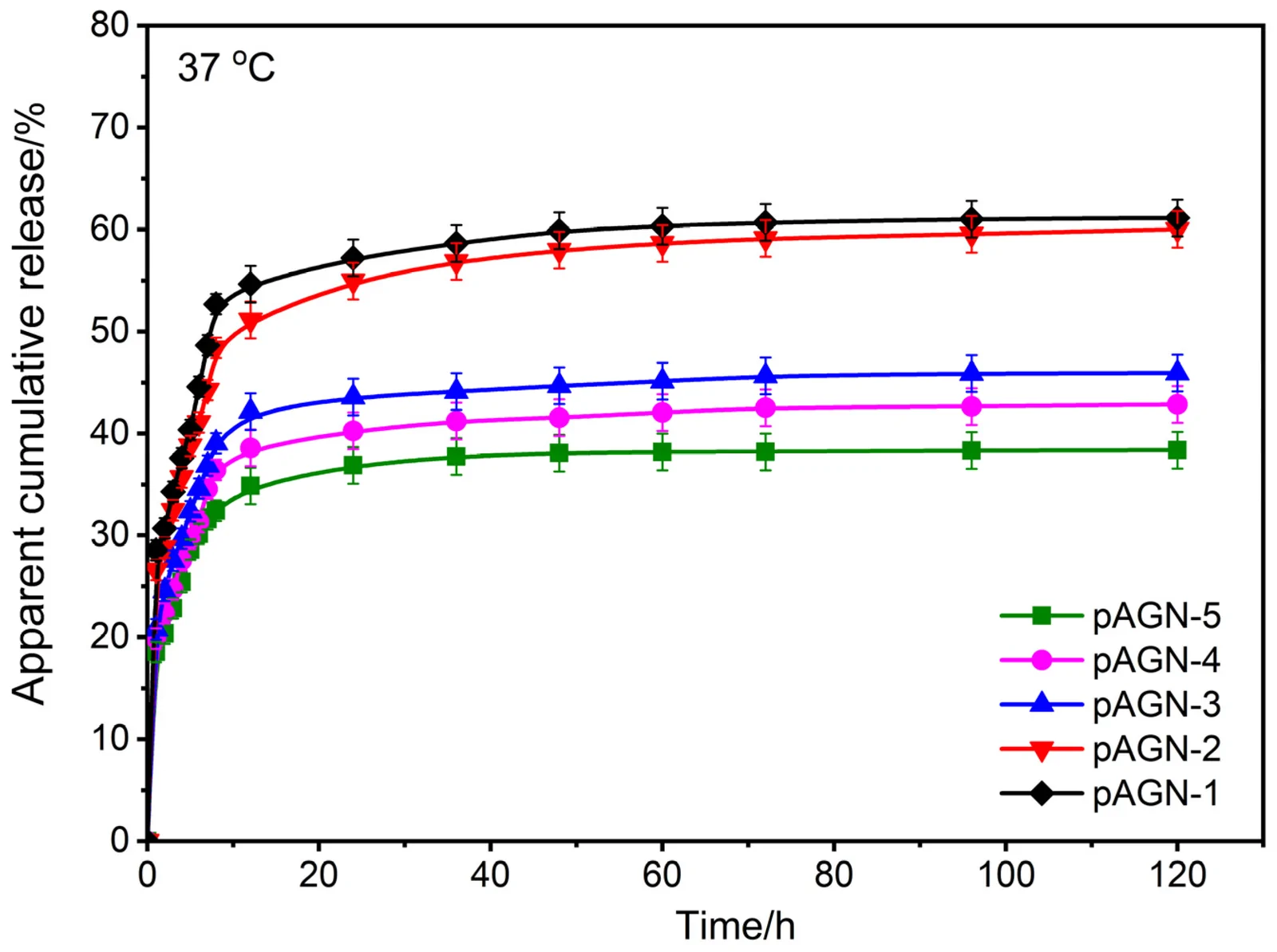
Apparent cumulative release curves of BSA from p(ASF-AGE-NIPAAm) hydrogels at 37 °C.

The in vitro BSA release profiles of the p(ASF-AGE-NIPAAm) hydrogels were investigated at two different temperatures (20 °C and 37 °C) to evaluate the thermoresponsive controlled-release characteristics of the prepared formulations. Figure 10 and Figure 11 present the apparent cumulative BSA release curves at 20 °C and 37 °C, respectively.

At 20 °C (below the LCST of all hydrogels), the release profiles (Figure 10) displayed a characteristic biphasic pattern for all five formulations. During the initial phase, the cumulative release rate increased rapidly, with all hydrogels achieving over 20% cumulative release within the first 8 h. This initial burst release is attributed to the highly hydrophilic and swollen state of the hydrogels at 20 °C, where BSA molecules located near the surface and within the loosely bound regions of the network are readily released through osmotic pressure-driven diffusion into the surrounding medium. Following this rapid initial release, a slower and more sustained release phase was observed between 12 h and 50 h, eventually approaching equilibrium at approximately 60 h. The final apparent cumulative release values at 20 °C were determined as follows: pAGN-1, 54.1%; pAGN-2, 51.0%; pAGN-3, 42.7%; pAGN-4, 40.7%; and pAGN-5, 37.6%. The sustained release during the later phase is likely dominated by BSA diffusion through the hydrated polymer network. However, polymer-chain relaxation may continue to contribute to the transport process, particularly in ASF-rich formulations. Therefore, the release behavior at 20 °C should be described as diffusion-dominated with relaxation contributions, rather than as a purely Fickian process. This interpretation is consistent with the non-Fickian swelling behavior observed in Section 2.3 [34].

At 37 °C (above the LCST of all hydrogels), markedly different release behavior was observed (Figure 11). Upon exposure to 37 °C, the apparent cumulative release increased rapidly, particularly for formulations with lower ASF content. For pAGN-1 and pAGN-2, the apparent cumulative release approached or exceeded 50% within the first 8 h, indicating substantially faster release than that observed at 20 °C. At the end of the release observation period, the apparent cumulative release values at 37 °C were 61.2%, 60.0%, 45.9%, 42.9%, and 38.4% for pAGN-1 to pAGN-5, respectively.

The accelerated release at 37 °C can be rationalized by the thermoresponsive phase transition behavior of the PNIPAAm component. At temperatures exceeding the LCST, the PNIPAAm chains undergo a hydrophilic-to-hydrophobic transition, resulting in rapid and substantial deswelling of the hydrogel network [35]. This thermally induced contraction generates internal hydrostatic pressure within the gel matrix, which acts as a driving force to expel both entrapped water molecules and BSA molecules from the collapsed network. The faster apparent release at 37 °C may be associated with thermally induced contraction of the PNIPAAm-rich network. Network contraction could facilitate BSA transport by reducing the hydrated volume and promoting the expulsion of water and solute. However, convective transport was not directly measured in the present study. Therefore, the release behavior at 37 °C is more appropriately described as diffusion with contributions from polymer-chain relaxation and contraction-associated solute expulsion [36].

A direct comparison of the release profiles below and above the LCST shows three points. The 20 °C condition lies below the LCST of all five hydrogels (31.8–33.0 °C), whereas the 37 °C condition lies above it. First, the release curves at 37 °C displayed steeper initial slopes within the first 8 h, indicating faster apparent release above the LCST. Second, the final apparent cumulative release values at 37 °C were consistently higher than those at 20 °C for all formulations, a difference consistent with temperature-induced network contraction. Third, hydrogels with higher ASF content (pAGN-4 and pAGN-5) exhibited lower apparent cumulative release than those with lower ASF content (pAGN-1 and pAGN-2) at both temperatures; this ASF-dependent trend is consistent with the lower swelling capacity and potentially greater transport resistance of ASF-rich hydrogels, and favorable ASF–BSA interactions may also contribute to BSA retention within the network [37]. Because the BSA concentrations in the release medium were below the lower limit of the reported calibration range, the cumulative release values are presented as apparent values and are used to compare temperature- and composition-dependent release trends under identical assay conditions.

The present results can be compared with previous silk fibroin/PNIPAAm studies and recent protein delivery platforms. Wang et al. reported regenerated ASF/PNIPAAm thermosensitive composite hydrogels with improved mechanical strength but did not evaluate protein loading or release [29]. Gil et al. prepared PNIPAAm/silk interpenetrating and macroporous hybrid hydrogels and examined their swelling/deswelling, rheological, and actuation behavior, again without protein delivery experiments [31,32]. Relative to our previous study, which established the synthesis, enzymatic degradability, and cytocompatibility of the p(ASF-AGE-NIPAAm) hydrogels [30], the present work adds equilibrium BSA loading, temperature-dependent apparent release, and kinetic analysis across five ASF-AGE compositions. A recent tea polyphenol nanoplatform was designed for cytosolic delivery of protein therapeutics [13], whereas the present hydrogels act as hydrated, temperature-responsive matrices for local protein retention and release. Because assay methods, protein cargos, and release environments differ among these systems, the comparison is made at the platform level rather than by direct numerical benchmarking.

BSA was selected as a representative model protein in this study; the present results should not be generalized directly to all therapeutic proteins. Proteins differ in molecular size, charge, isoelectric point, hydrophobicity, conformational stability, and aggregation tendency, and each of these factors can affect partitioning, diffusion, and interactions with ASF and PNIPAAm. Future studies should test representative therapeutic proteins with different physicochemical properties and assess protein integrity and bioactivity after loading and release.

### 2.10. Drug Release Mechanism

To elucidate the underlying drug release mechanism of the p(ASF-AGE-NIPAAm) hydrogels, three well-established kinetic models, namely the first-order model, the Higuchi model, and the Korsmeyer–Peppas model, were employed to fit the experimental release data. The mathematical expressions of these models are presented in Table 4.

The first-order model assumes that the release rate decreases in proportion to the amount of BSA remaining in the hydrogel, and therefore describes release processes governed by progressive drug depletion. The Higuchi model describes diffusion-controlled release from a homogeneous matrix under sink conditions, assuming relatively constant matrix geometry and diffusion coefficient. The Korsmeyer–Peppas model is an empirical model in which the exponent n is used to distinguish diffusion-controlled, anomalous, and relaxation-controlled transport. Because p(ASF-AGE-NIPAAm) hydrogels undergo hydration, polymer-chain relaxation, and temperature-dependent contraction, these models were used for comparative analysis rather than as definitive proof of a single release mechanism.

**Figure 12:**
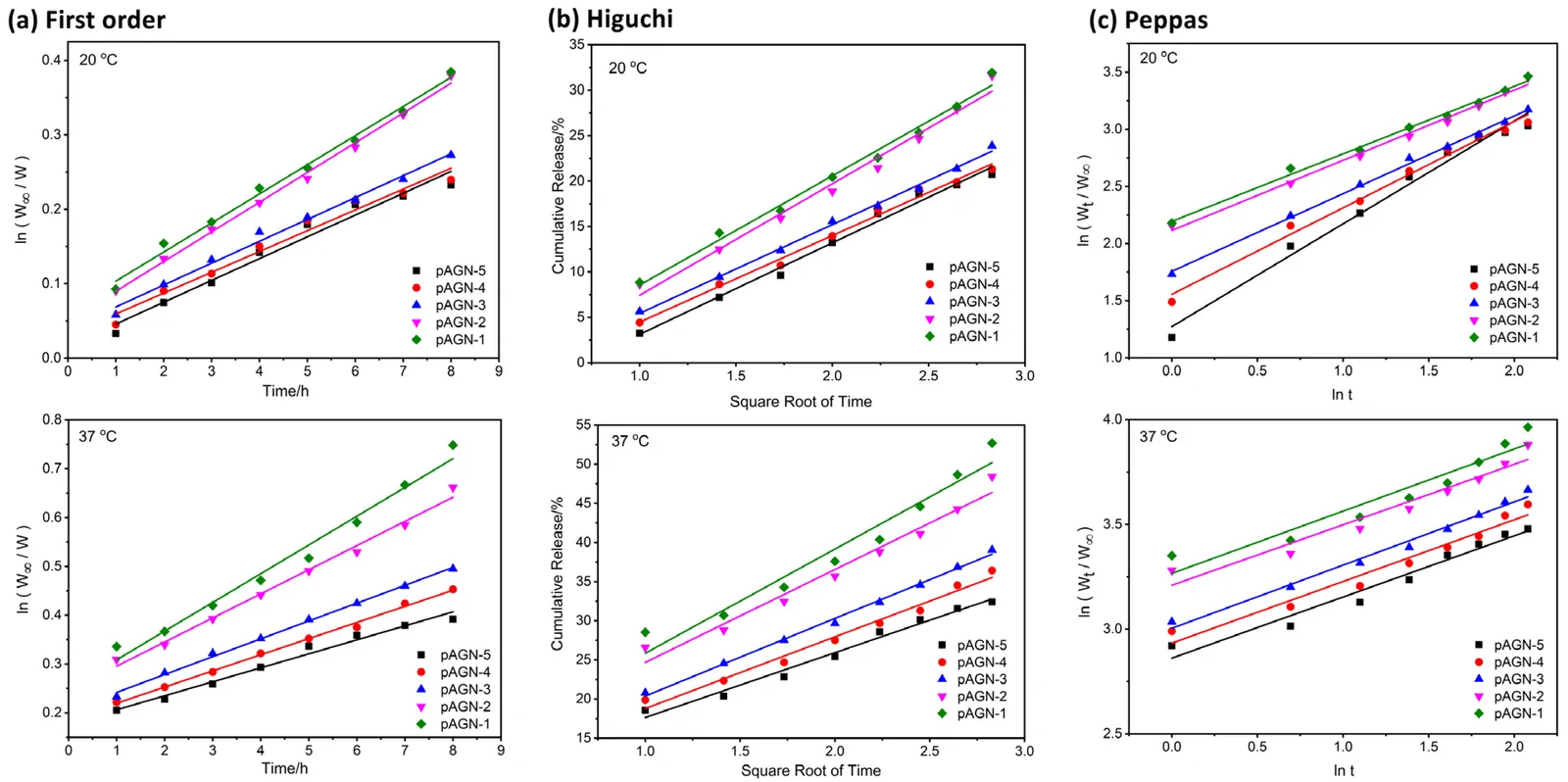
Fitting curves of the BSA release data with three kinetic models: (**a**) first-order model; (**b**) Higuchi model; (**c**) Korsmeyer–Peppas model.

The fitting results of the three kinetic models are shown in Figure 12 and Table 5. The first-order and Higuchi models both correlated well with the experimental data, with R^2^ values exceeding 0.94 for all formulations at both temperatures, and the Korsmeyer–Peppas model gave R^2^ values between 0.910 and 0.992 together with the diffusion exponent n for each dataset. The AIC values in Table 5 support this composition- and temperature-dependent assignment: the model with the highest R^2^ also gave the lowest AIC for eight of the ten datasets, and for the remaining two datasets (pAGN-1 at 20 °C and pAGN-5 at 37 °C) the AIC difference between the two top-ranking models is smaller than 2, indicating that the two models describe these release data comparably well.

Both the first-order and Higuchi models provided good correlations with the experimental release data, with R^2^ values exceeding 0.94 for all formulations at both temperatures. However, the best-fitting model was dependent on hydrogel composition and temperature. At 20 °C, the Higuchi model provided higher R^2^ values for pAGN-3, pAGN-4, and pAGN-5, whereas the first-order model provided better fits for pAGN-1 and pAGN-2. At 37 °C, the first-order model gave higher R^2^ values for pAGN-1 to pAGN-4, while the Higuchi model remained slightly superior for pAGN-5. Therefore, the release profiles should not be assigned to a single kinetic model for all formulations.

It should be emphasized that crosslinking density, pore size, ASF–PNIPAAm hydrogen-bond density, and β-sheet content were not directly measured in the present study. Therefore, the structural explanations proposed above are inferred from composition-dependent swelling, deswelling, BSA loading, and release behavior, together with the known structural characteristics of ASF reported in the literature [28,30].

AIC was calculated as AIC = *n* ln(SSR/*n*) + *2p* from the residual sum of squares (SSR) of each fit on the original (untransformed) release-percentage scale, with *n* = 8 data points in the 1–8 h fitting interval and p = 2 fitted parameters for each model; the small-sample correction AICc = AIC + 2.4 is identical for all models and does not change the ranking. For pAGN-1 at 20 °C and pAGN-5 at 37 °C, the AIC values of the two top-ranking models differ by less than 2, so the two fits are statistically comparable.

Although the Higuchi model was not universally the best-fitting model, its rate constant (k) remains useful for comparing the relative release behavior of the different formulations. Overall, the k values decreased with increasing ASF content at both temperatures: the two formulations with the lowest ASF loading (pAGN-1 and pAGN-2) gave the highest k values (11.88–13.32), whereas the two formulations with the highest ASF loading (pAGN-4 and pAGN-5) gave the lowest (8.29–10.07). This trend is consistent with the formation of a more constrained and less permeable network at higher ASF loadings, which may restrict polymer-chain mobility and increase the tortuosity of BSA diffusion pathways [38]. The temperature dependence of k was not uniform across formulations: k was higher at 37 °C than at 20 °C for pAGN-1 and pAGN-3 but slightly lower for pAGN-2, pAGN-4, and pAGN-5. This arises because the 37 °C profiles show a larger initial burst within the first hour (Figure 11), which raises the intercept and lowers the slope of the Higuchi fit over the 1–8 h interval; the thermally induced network contraction above the LCST therefore appears mainly as an enhanced early burst rather than a larger Higuchi slope [39]. Because crosslinking density and pore size were not directly measured in the present study, these structural interpretations should be regarded as inferences from the swelling and release behavior.

The Korsmeyer–Peppas model provided additional empirical information on the release behavior. The diffusion exponent (*n*) can be used to distinguish between different transport mechanisms: *n* ≤ 0.5 indicates Fickian diffusion, 0.5 < *n* < 1.0 indicates anomalous or non-Fickian transport involving both diffusion and polymer-chain relaxation, and *n* = 1.0 indicates Case-II transport [34]. At 20 °C, the *n* values ranged from 0.591 to 0.901, consistent with anomalous transport involving both diffusion and polymer relaxation. At 37 °C, the *n* values ranged from 0.289 to 0.302. However, because the hydrogel undergoes contraction and changes in hydration during release at this temperature, these exponents should be interpreted cautiously and should not be used alone to assign a purely Fickian release mechanism.

The good Higuchi correlations suggest that diffusion through the hydrated polymer matrix contributes substantially to BSA release [34]. Meanwhile, the improved first-order correlations, particularly at 37 °C, indicate that the release rate may also depend on the amount of BSA remaining in the hydrogel and on contraction-assisted expulsion during thermally induced deswelling. Because the hydrogel volume, water content, and effective diffusion coefficient change during the phase transition, the kinetic fitting results should be regarded as empirical descriptions rather than definitive proof of a single release mechanism. Taken together, the release behavior is more appropriately described as a coupled process involving BSA diffusion, polymer-chain relaxation, and temperature-induced network contraction.

## 3. Conclusions

In this study, a previously developed p(ASF-AGE-NIPAAm) hydrogel platform was systematically evaluated for protein drug delivery using BSA as a model protein. Increasing the ASF-AGE feed content reduced the equilibrium swelling ratio and slightly decreased the LCST from 33.0 °C to 31.9 °C. The hydrogels exhibited high BSA encapsulation efficiencies of 73.5–76.8%, whereas the drug loading capacity decreased from 453 to 380 mg/g with increasing ASF-AGE content. The apparent BSA release behavior was dependent on both temperature and formulation, with higher apparent cumulative release at 37 °C than at 20 °C and lower release generally observed for ASF-rich formulations.

Kinetic analysis showed that the best-fitting model depended on formulation and temperature, indicating that the release profiles should not be assigned to a single kinetic model. The release behavior is more appropriately described as an empirical, coupled process involving BSA diffusion, polymer-chain relaxation, and contraction-associated solute transport. The main contribution of this study is the establishment of a composition-property-release relationship for the p(ASF-AGE-NIPAAm) platform, which may guide the future optimization of ASF/PNIPAAm-based temperature-responsive protein delivery carriers. Further studies should include low-concentration calibration or a more sensitive protein assay, direct structural characterization, evaluation of additional therapeutic proteins, and in vivo validation. Recent functional hydrogel research illustrates how network engineering and intermolecular design can integrate mechanical, sensing, and biological functions [40]; applying similar principles to ASF/PNIPAAm systems will support their further development toward practical protein delivery applications.

Beyond these laboratory-scale findings, translating this platform toward practical protein delivery will require systematic evaluation of large-scale fabrication, batch-to-batch reproducibility, sterilization, storage stability, in vivo degradation behavior, and immunological response, together with the preservation of protein bioactivity throughout processing and storage. Standardized structural characterization and validated protein assays will also be essential for regulatory assessment and for benchmarking against other delivery systems.

## 4. Materials and Methods

### 4.1. Materials

Disodium hydrogen phosphate (Na_2_HPO_4_, analytical grade) and potassium dihydrogen phosphate (KH_2_PO_4_, analytical grade) were purchased from Sinopharm Chemical Reagent Co., Ltd. (Shanghai, China) and used for preparing phosphate-buffered saline (PBS). Absolute ethanol (analytical grade) was obtained from Sinopharm Chemical Reagent Co., Ltd. (Shanghai, China). Coomassie Brilliant Blue R250 (analytical grade) was supplied by Fuzhou PHYGENE Scientific Co., Ltd. (Fuzhou, China). Bovine serum albumin (BSA, biological reagent grade) was purchased from Fuzhou PHYGENE Scientific Co., Ltd. (Fuzhou, China) and used as the model protein drug. All chemicals were used as received without further purification unless otherwise noted. Deionized water was used throughout all experiments.

### 4.2. Synthesis of p(ASF-AGE-NIPAAm) Hydrogels

Allyl silk fibroin (ASF-AGE) was obtained through nucleophilic substitution between regenerated Antheraea pernyi silk fibroin (ASF) and allyl glycidyl ether (AGE) according to our previously reported method [30]. The p(ASF-AGE-NIPAAm) hydrogels were synthesized following the procedure reported previously [30]. Briefly, NIPAAm was purified by recrystallization in a toluene/n-hexane mixture (6:4, *v*/*v*). ASF-AGE was dissolved in deionized water (5% *w*/*v*), mixed with ammonium persulfate (APS) and N,N,N′,N′-tetramethylethylenediamine (TEMED) in an ice bath, and then NIPAAm solution (75 mg/mL) was added dropwise to the mixture under continuous nitrogen flow at 20 °C. After polymerization, the viscous solution was injected into a glass mold, bubbled with nitrogen for 30 min, sealed with a Teflon stopper, and incubated at room temperature for 12 h. The obtained hydrogels were dialyzed against deionized water for 2 days (8–14 kDa molecular weight cutoff) to remove unreacted chemicals. The five formulations with varying ASF-AGE:PNIPAAm mass ratios (1:9, 1:4, 1:2.5, 1:1.5, and 1:0.75) were denoted as pAGN-1 to pAGN-5, respectively (Table 6). The drug-free hydrogels thus obtained were used directly as the blank samples for the swelling, deswelling, temperature-responsiveness, and pulsatile-response measurements described below.

### 4.3. Swelling Ratio (SR) Determination

The dynamic swelling behavior of the hydrogels was investigated using a gravimetric method. Pre-weighed dry hydrogel samples (mass = M_d_) were immersed in 0.03 M PBS (pH 7.4) at 20 °C. At predetermined time intervals, the samples were removed from the swelling medium, gently blotted with filter paper to remove excess surface water, and immediately weighed (M_t_). PBS at this concentration provides stable buffering at pH 7.4 with an ionic strength close to physiological conditions, and the same medium was used in our previous study of this platform [30] to allow direct comparison. The swelling ratio (SR) was calculated according to the following equation:

(4) $$SR(g/g)=\frac{M_{t}-M_{d}}{M_{d}}$$

where M_t_ is the mass of the swollen hydrogel at time t, and M_d_ is the mass of the dry hydrogel. Each experiment was performed in triplicate, and the results are expressed as mean values.

### 4.4. Equilibrium Swelling Ratio (ESR) Measurement

To investigate the temperature-dependent swelling behavior, pre-weighed dry hydrogels were placed in 0.03 M PBS (pH 7.4) at various temperatures ranging from 15 °C to 50 °C (in 5 °C increments) and allowed to equilibrate until no further weight change was observed. The ESR was calculated using the same equation as for the swelling ratio. The ESR data as a function of temperature were used to determine the LCST of the hydrogels.

### 4.5. LCST Determination

The LCST was determined from the equilibrium swelling data obtained at different temperatures. The ESR versus temperature curves were plotted, and the first-order derivative of the ESR with respect to temperature (d(ESR)/d(T)) was calculated. The peak temperature corresponding to the maximum absolute value of the first-order derivative was identified as the LCST of the hydrogel.

### 4.6. Deswelling Studies

The deswelling kinetics were evaluated to assess the response rate of the hydrogels upon temperature increase above the LCST. Hydrogel samples were first equilibrated in PBS at a temperature below the LCST to reach the fully swollen state (equilibrium mass = Me). The swollen samples were then rapidly transferred to a water bath maintained at 40 °C (above the LCST). At predetermined time intervals, the samples were removed, surface water was blotted, and the residual swollen mass (M_t_) was recorded. The water loss rate (WLR) was calculated as follows:

(5) $$WLR\%=\frac{M_{e}-M_{t}}{M_{e}-M_{d}}\times 100\%$$

where M_e_ is the equilibrium swollen mass, M_t_ is the mass at time t, and M_d_ is the dry mass. The WR (in %) was subsequently calculated as:

(6) WR(%)=100%−WLR%

### 4.7. Reversible Swelling–Deswelling (Pulsatile Response) Studies

The reversible temperature responsiveness of the hydrogels was investigated through cyclic swelling–deswelling experiments. Hydrogel samples were alternately immersed in PBS at 20 °C (below the LCST) and 40 °C (above the LCST) for successive cycles. The swelling ratio was measured every 20 min by the gravimetric method described in Section 4.3. This temperature-switching procedure was repeated for multiple cycles to evaluate the reproducibility and stability of the thermally induced volume phase transition.

### 4.8. BSA Quantification and Calibration

BSA concentrations were estimated using the Coomassie Brilliant Blue R250 assay. Briefly, 1.0 mL of BSA standard or sample was mixed with 3.0 mL of Coomassie Brilliant Blue R250 reagent, and the absorbance was measured at 595 nm using a spectrophotometer (UV3600, Shimadzu, Japan). OD595 was defined as the absorbance at 595 nm. BSA calibration standards with concentrations ranging from 1 to 9 mg/mL were prepared in 0.03 M PBS and analyzed under the same assay conditions as the samples. The calibration equation was y = 0.1024 + 0.0182x, where y is OD595 and x is the BSA concentration in mg/mL. Unknown BSA concentrations were calculated as x = (y − 0.1024)/0.0182. The same buffer, assay procedure, and calibration equation were used for all loading and release samples to ensure consistency in the relative comparison among different hydrogel formulations.

### 4.9. Drug Loading

Pre-weighed dry hydrogel samples (1 cm × 1 cm) were immersed in 10 mL of BSA solution (5 mg/mL in 0.03 M PBS) and incubated at 25 °C for 5 days under gentle shaking to allow equilibrium loading. At the end of the loading period, the residual BSA concentration in the supernatant was determined by the Coomassie Brilliant Blue R250 method at 595 nm using the established standard curve. The amount of BSA loaded into the hydrogel was calculated by subtracting the residual BSA amount from the initial total amount.

The encapsulation efficiency (EE) and drug loading capacity (DL) were calculated according to the following equations:

(7) $$\mathrm{EE}\left(\%\right)=\frac{\mathrm{Total}\;\mathrm{BSA}-\mathrm{Residual}\;\mathrm{BSA}}{\mathrm{Total}\;\mathrm{BSA}}\times 100\%$$

(8) $$\mathrm{DL}\left(mg/g\right)=\frac{\mathrm{Mass}\;\mathrm{of}\;\mathrm{loaded}\;\mathrm{BSA}}{\mathrm{Mass}\;\mathrm{of}\;\mathrm{dry}\;\mathrm{hydrogel}+\mathrm{Mass}\;\mathrm{of}\;\mathrm{loaded}\;\mathrm{BSA}}$$

Each drug loading experiment was performed in triplicate.

### 4.10. In Vitro Drug Release

The in vitro BSA release profiles were evaluated at two different temperatures: 20 °C (below the LCST) and 37 °C (above the LCST). BSA-loaded hydrogel samples were placed in 50 mL of 0.03 M PBS (pH 7.4) in a thermostatic shaking incubator at a rotation speed of 50 rpm. At predetermined time intervals, 0.2 mL of the release medium was withdrawn and replaced with an equal volume of fresh PBS to maintain sink conditions. The collected samples were stained with Coomassie Brilliant Blue R250, and the absorbance at 595 nm was measured to determine the BSA concentration.

The cumulative amount of BSA released (W) was calculated using the following equation:

(9) $$W=50\times C_{t}+\sum C_{t-1}\times 0.2$$

where C_t_ is the BSA concentration (mg/mL) in the release medium at time t, 50 is the total volume (mL) of the release medium, and the summation term (ΣC_t − 1_ × 0.2) accounts for the BSA removed in the previously withdrawn 0.2 mL samples. The cumulative release percentage was then calculated as:

(10) $$\mathrm{Release}\left(\%\right)=\frac{W}{W_{\infty }}\times 100\%$$

where W_∞_ is the total amount of BSA initially loaded in the hydrogel. The apparent cumulative release data were fitted to the first-order, Higuchi, and Korsmeyer–Peppas models as empirical descriptions of the release behavior. The model equations and fitting parameters are presented in Section 2.10.

### 4.11. Statistical Analysis

All experiments were performed in triplicate (*n* = 3). For each formulation, three hydrogel specimens taken from the same synthesis batch were measured independently, and the results are reported as mean ± standard deviation (SD). Curve fitting and data plotting were performed using OriginPro (OriginLab, Northampton, MA, USA). The coefficient of determination (R^2^) and the Akaike information criterion (AIC) were used to compare the kinetic models, with lower AIC values indicating a better balance between goodness of fit and model complexity.

### 4.12. Statement of Generative AI Tool Usage

In this study, the generative AI tool Kimi (K3) was used as an auxiliary editing tool during the final polishing phase of the manuscript. Its application was strictly limited to manuscript formatting optimization and language verification, and it did not participate in any core research processes. Generative AI tools were employed to proofread the English manuscript for spelling and grammatical errors, standardize academic English phrasing, verify the completeness of reference entries, and unify citation formats and ordering conventions.

## Figures and Tables

**Table 1:** Swelling kinetic parameters of the p(ASF-AGE-NIPAAm) hydrogels.

Sample	*n* Value	R^2^
pAGN-1	0.596	0.999
pAGN-2	0.678	0.999
pAGN-3	0.717	0.992
pAGN-4	0.712	0.992
pAGN-5	0.821	0.998

**Table 2:** Deswelling kinetic parameters of the p(ASF-AGE-NIPAAm) hydrogels.

Sample	k Value	R^2^
pAGN-1	−0.0256	0.936
pAGN-2	−0.0283	0.937
pAGN-3	−0.0274	0.953
pAGN-4	−0.0301	0.964
pAGN-5	−0.0281	0.988

**Table 3:** Encapsulation efficiency and drug loading capacity of p(ASF-AGE-NIPAAm) hydrogels for BSA.

Sample	EE (%)	DL (mg/g)
pAGN-1	73.5 ± 1.3	453 ± 14
pAGN-2	73.9 ± 1.4	445 ± 13
pAGN-3	73.6 ± 1.3	421 ± 15
pAGN-4	74.3 ± 1.6	395 ± 17
pAGN-5	76.8 ± 1.3	380 ± 17

**Table 4:** Kinetic models of BSA release.

Kinetic Model	Equation
First-order	y = 100(1 − e^−kt^)
Higuchi model	y = kt^1/2^
Korsmeyer–Peppas model	y = kt^n^

**Table 5:** Fitting parameters of the three kinetic models for BSA release.

Temperature	Sample	Best Fit Model	First-Order			Higuchi Model			Korsmeyer–Peppas Model		
			R^2^	k	AIC	R^2^	k	AIC	R^2^	n	AIC
20 °C	pAGN-5	Higuchi	0.966	0.029	3.55	0.988	10.07	−3.82	0.983	0.900	6.05
37 °C	pAGN-5	Higuchi	0.979	0.029	−3.36	0.980	8.29	−3.06	0.957	0.292	1.44
20 °C	pAGN-4	Higuchi	0.969	0.028	2.08	0.990	9.52	−6.77	0.987	0.758	0.29
37 °C	pAGN-4	First-order	0.996	0.033	−15.45	0.980	9.16	−1.27	0.956	0.295	4.96
20 °C	pAGN-3	Higuchi	0.989	0.029	−5.11	0.996	9.76	−13.62	0.992	0.680	−12.97
37 °C	pAGN-3	First-order	0.997	0.037	−13.78	0.996	9.92	−12.77	0.985	0.302	−1.83
20 °C	pAGN-2	First-order	0.996	0.040	−10.02	0.983	12.27	3.14	0.981	0.614	0.45
37 °C	pAGN-2	First-order	0.990	0.050	−3.46	0.968	11.88	6.50	0.937	0.289	11.88
20 °C	pAGN-1	First-order	0.991	0.039	−3.29	0.988	12.03	−1.16	0.983	0.591	−3.37
37 °C	pAGN-1	First-order	0.979	0.059	5.53	0.948	13.32	12.43	0.910	0.297	16.82

**Table 6:** Formulations for the synthesis of p(ASF-AGE-NIPAAm) hydrogels.

Hydrogel Code	ASF-AGE (mg)	NIPAAm (mL)	ASF-AGE: NIPAAm (Mass Ratio)	APS (mg)	5% TEMED (µL)
pAGN-1	50.0	6.0	1:9	9.5	60
pAGN-2	100.0	5.33	1:4	7.9	60
pAGN-3	142.9	4.76	1:2.5	7.1	60
pAGN-4	200	4.0	1:1.5	6.0	60
pAGN-5	285.7	2.86	1:0.75	4.3	60

## Data Availability

The data presented in this study are available on request from the corresponding author.

## Abbreviations

The following abbreviations are used in this manuscript:

ASF	*Antheraea pernyi* silk fibroin
AGE	Allyl glycidyl ether
NIPAAm	N-isopropylacrylamide
PNIPAAm	Poly(N-isopropylacrylamide)
LCST	Lower critical solution temperature
BSA	Bovine serum albumin
PBS	Phosphate-buffered saline
SR	Swelling ratio
WR	water retention
ESR	Equilibrium swelling ratio
WLR	Water loss rate
EE	Encapsulation efficiency
DL	Drug loading
